# Dysregulated CD4+ T Cells and microRNAs in Myocarditis

**DOI:** 10.3389/fimmu.2020.00539

**Published:** 2020-03-25

**Authors:** Jing Wang, Bo Han

**Affiliations:** Department of Pediatric Cardiology, Shandong Provincial Hospital Affiliated to Shandong University, Jinan, China

**Keywords:** CD4+ T cells, microRNA, myocarditis, experimental myocarditis, pathogenesis, diagnosis, therapy

## Abstract

Myocarditis is a polymorphic disease complicated with indeterminate etiology and pathogenesis, and represents one of the most challenging clinical problems lacking specific diagnosis and effective therapy. It is caused by a complex interplay of environmental and genetic factors, and causal links between dysregulated microribonucleic acids (miRNAs) and myocarditis have also been supported by recent epigenetic researches. Both dysregulated CD4+ T cells and miRNAs play critical roles in the pathogenesis of myocarditis, and the classic triphasic model of its pathogenesis consists of the acute infectious, subacute immune, and recovery/chronic myopathic phase. CD4+ T cells are key pathogenic factors underlying the development and progression of myocarditis, and the effector and regulatory subsets, respectively, promote and inhibit autoimmune responses. Furthermore, the reciprocal interplay of these subsets influences the pathogenesis as well. Dysregulated miRNAs along with their mRNA and protein targets have been identified in heart biopsies (intracellular miRNAs) and body fluids (circulating miRNAs) during myocarditis. These miRNAs show phase-dependent changes, and correlate with viral infection, immune status, fibrosis, destruction of cardiomyocytes, arrhythmias, cardiac functions, and outcomes. Thus, miRNAs are promising diagnostic markers and therapeutic targets in myocarditis. In this review, we review myocarditis with an emphasis on its pathogenesis, and present a summary of current knowledge of dysregulated CD4+ T cells and miRNAs in myocarditis.

## Introduction

Myocarditis is the inflammation of the myocardium, and a relatively common but potentially life-threatening disease that affects millions globally, especially pediatric patients and young adults, though it is difficult to gauge the incidence rates (1–7). Despite the well-established definition, the real-world myocarditis represents one of the most challenging clinical problems lacking specific diagnosis and effective therapy, and current state of knowledge on its etiology and pathogenesis remains poorly understood (1, 2). Myocarditis is the result of a complex interplay of environmental and genetic factors (2). In addition, causal links between dysregulated microribonucleic acids (miRNAs) and myocarditis have also been supported by recent epigenetic studies (8). Based on clinical and animal data, the classic triphasic model of pathogenesis of myocarditis was proposed, which consists of the acute infectious, subacute immune, and recovery/chronic myopathic phase (2–4, 8, 9), and both dysregulated CD4+ T cells and miRNAs play critical roles.

CD4+ T cells are key pathogenic factors underlying the development and progression of myocarditis, and the effector and regulatory subsets, respectively, promote and inhibit autoimmune responses. T helper (Th) 1 cells initiate tissue damage, promote early responses, and protect against excessive cardiac inflammation (8, 10, 11). Th 2 cells are critical in severe myocarditis with eosinophil expansion (12, 13). Th17 cells are major regulators in chronic phase (8, 14, 15). In contrast, regulatory T (Treg) cells relieve acute cardiac inflammation and prevent progression from myocarditis to dilated cardiomyopathy (DCM) (16–21). Furthermore, the reciprocal interplay of these subsets influences the pathogenesis of myocarditis as well, and roles of dysregulated miRNAs modulating CD4+ T cells in the pathogenesis of myocarditis have gained attention recently.

miRNAs are endogenous non-coding small RNA molecules that post-transcriptionally fine-tune their target genes. They are critical epigenetic regulators of cardiac function, take part in almost all aspects of cardiac physiology and pathology (22), and are involved in both the etiology and pathogenesis of myocarditis. With the development of molecular techniques in recent years, miRNA profiles of myocarditis have been analyzed (23), and dysregulated miRNAs along with their mRNA and protein targets have been identified in heart biopsies (intracellular miRNAs) and body fluids (circulating miRNAs). The former in this review are further and roughly classified into myomiRs, cardiotropic viral infection-related miRNAs, immune status-related miRNAs, cardiotropic viral infection and immune status -related miRNAs, fibrosis-related miRNAs, and miscellaneous miRNAs. Dysregulated miRNAs in myocarditis show phase-dependent changes, and correlate with viral infection, immune status, fibrosis, destruction of cardiomyocytes, arrhythmias, cardiac functions, and outcomes. Thus, miRNAs are promising diagnostic markers and therapeutic targets in myocarditis.

In this review, we provide a brief overview of myocarditis with an emphasis on its pathogenesis, and then summarize dysregulated CD4+ T cells subsets and the reciprocal interactions of these subsets in myocarditis. As miRNAs are involved in both the etiology and pathogenesis of myocarditis, we proceed to review the biogenesis and function of miRNAs, and discuss miRNAs that modulate CD4+ T cells. Furthermore, we gather results published on miRNAs expression profiles in myocarditis and select representative miRNAs to briefly highlight the regulatory mechanisms, as well as the diagnostic and therapeutic potential of these miRNAs in myocarditis. Finally, some present perspectives and future directives for myocarditis are suggested.

## Myocarditis: A Brief Overview

### Definition, Clinical Presentation, Diagnosis, Prevalence, Prognosis, and Therapy

Myocarditis is the inflammatory condition of the myocardium and defined by certain histological, immunological, and immunohistochemical criteria (2). According to the Dallas criteria, myocarditis is histologically defined as the presence of inflammatory infiltrates within the myocardium in conjunction with non-ischemic damage to adjacent myocytes (24). The ESC working group immunologically characterizes myocarditis as ≥14 leucocytes/mm^2^ including up to 4 monocytes/mm^2^ along with CD3^+^ T-lymphocytes ≥7 cells/mm^2^ (2).

Despite these well-established criteria, the real-world myocarditis is a polymorphic disease with clinical presentations varying from non-specific systemic symptoms to severe life-threatening scenarios such as acute heart failure, malignant arrhythmia, or even sudden death (1). In most cases, clinical presentations of patients with biopsy-confirmed myocarditis (excluding coronary artery diseases or other known causes) can be classified as: (a) acute coronary syndrome-like, (b) heart failure, and (c) arrhythmias (1, 2).

It is often challenging to diagnose myocarditis due to non-specific symptoms, limitations of current diagnostic strategies, and evolving pathogenesis (1). Currently, the diagnostic gold standard is endomyocardial biopsy which can characterize the disease on histological, immunological, and molecular parameters (2, 3). However, the fairly high false negative rates due to focal inflammation and inter-observer variability obviate definite diagnosis of myocarditis. Therefore, a combination of medical history, clinical assessment, myocardial biochemical markers, and imaging (such as electrocardiograms, echocardiograms, chest X-rays, cardiac magnetic resonance imaging) results should be considered to confirm suspected myocarditis. However, the currently available non-invasive myocardial biochemical markers and predictive methods lack sufficient accuracy and therefore cannot be generally recommended in clinical practice. Since miRNAs display phase-dependent changes in myocarditis, and correlate with disease severity, they are potential diagnostic markers and may be useful to predict disease outcomes.

Relatively low diagnosis rates translate to poorly documented epidemiology leading to underestimation of the global incidence rates. Nevertheless, myocarditis is a relatively common but potentially life-threatening disease that affects millions globally, especially pediatric patients and young adults (1–7). According to the Global Burden of Disease Study, the most recently reported age-standardized rate of myocarditis was 19.1 cases per 100,000 patients in 2016, a reduction of 5.7% compared to that in 1990 (25). The incidence rates may be underestimated since many patients with clinical manifestations of myocarditis do not undergo endomyocardial biopsy (26). In addition, a retrospective review of 17,162 postmortem records found that myocarditis is often overlooked by clinicians and affects young adults more frequently (6). Similarly, biopsy-proven myocarditis is reported in 46% of children with an identified cause of DCM, which is nearly 3–5 times higher than that in adult patients (2, 7).

Although ~40–66% of the cases resolve spontaneously and recover completely in the first 4–12 weeks (27), myocarditis can progress to stable DCM with phenotypic characteristics like dilated chamber, decreased myocardial contractility, stiffened chamber, and/or arrhythmia in susceptible individuals. Patients with DCM frequently develop heart failure with high mortality (8), and studies addressing biopsy-proven myocarditis in patients with DCM report highly variable prevalence ranging from 9 to 30% (2, 3, 6, 28).

Currently, the treatment of patients with myocarditis is mainly supportive and rather empirical, and the core principles of therapies of myocarditis are optimal care of arrhythmia, heart failure, and etiology-targeted therapy (2). There are neither guidelines dedicated to myocarditis nor strategies to tailor therapies to patient's most favorable benefit (29). As such, there is an urgent need to focus on the etiology and pathogenesis of myocarditis to identify effective diagnostic biomarkers and therapeutic targets for the treatment and prevention of myocarditis.

### Etiology and Experimental Myocarditis

Current state of knowledge on etiology and pathogenesis of myocarditis remains poorly investigated. A complex interplay of environmental and genetic factors is responsible for myocarditis (2). In addition, causal links between dysregulated miRNAs and myocarditis have also been supported by recent epigenetic studies. Ablation of total miRNAs results in severe developmental defects in the cardiovascular system (30, 31), and most pathological conditions are associated with multiple aberrantly expressed miRNAs (31, 32). Despite the indeterminate etiology, myocarditis can be classified into the infectious or non-infectious types. Infectious myocarditis is caused by pathogens such as viruses, bacteria, spirochetes, fungi, parasites, and rickettsia, while non-infectious myocarditis results from immunological factors like allergens and allo-/auto-antigens, toxins including drugs, heavy metals, hormones, physical agents etc., and miscellaneous factors like animal bites (2). The most frequent cause of myocarditis in developed countries is viral infections, however, rheumatic carditis and bacterial infections predominate in the developing world (4), and the endemic parasite *Trypanosoma cruzi* is the primary causative agent in Latin America (33).

To elucidate its underlying cellular and molecular mechanisms, animal (such as rodent, porcine, and canine models) and cell (such as cardiomyocyte HL-1 cells, cardiomyocyte H9c2 cells) models of myocarditis have been developed. These animal models can be experimentally induced to simulate the multiple etiologies of human myocarditis (8, 34–37). Pathogens that induce myocarditis in humans such as coxsackievirus B3 (CVB3), lipopolysaccharide (LPS) and *Trypanosoma cruzi*, et al. are used to establish the infectious models. Meanwhile, cardiac antigens such as α-isoform of myosin heavy chain or troponin I peptide, either delivered with a strong adjuvant or within dendritic cells (DCs), are typically used to trigger the autoimmune response in non-infectious models.

### Pathogenesis of Myocarditis

**Figure 1:**
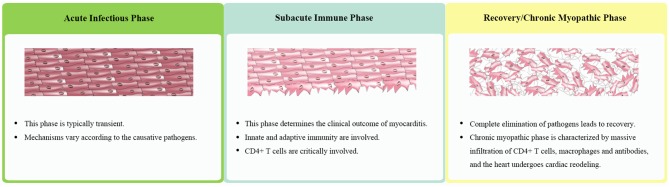
The triphasic model of pathogenesis of myocarditis.

Taking together clinical observations as well as data from animal models, the classic triphasic model of pathogenesis of myocarditis was proposed, which consists of the acute infectious, subacute immune, and recovery/chronic myopathic phase (2–4, 8, 9) (Figure 1).

#### Acute Infectious Phase

The acute infectious phase is typically transient and often overlooked by clinicians. The mechanisms of acute myocardial injury and the accompanying inflammatory mediators and cytokines vary according to causative pathogens. In virus-induced myocarditis for example, viral entry into the myocardium can not only cause direct injury by producing inflammatory mediators such as type 1 interferons, and inducing apoptosis and autophagy (38), but also indirectly damage the tissues by triggering an immune response. In contrast, myocarditis occurring with Chagas disease involves the interplay of multiple inflammatory cells, bioactive compounds released by parasites, and oxidative stress (39).

#### Subacute Immune Phase

The state of immune response determines the clinical outcome of myocarditis. While an appropriate immune response can eliminate pathogens, overactivation can cause excessive tissue damage resulting in organ dysfunction. CD4+ T cells are critically involved in the immune phase, and typically peak during the first 1–2 weeks coinciding with the most severe clinical phase of disease (9).

##### Innate immunity

Cardiac inflammation and tissue injury activate the innate immune response. Most cardiac cells constitutively express innate immune receptors like the Toll-like receptors (TLRs) that recognize and bind to specific molecular patterns on pathogens (40). Once engaged, receptors transmit a cascade of signals to activate nuclear transcription factors such as nuclear factor-κB (NF-κB) leading to the production of pro-inflammatory cytokines (41, 42). In addition, they can also induce pyroptosis and adaptive immune system consequently (43, 44).

##### Adaptive immunity

The adaptive immune response is initiated when T cell receptors (TCRs) bind to antigen epitopes presented on MHC molecules (45, 46), and then participates in myocarditis via activation of T cells and B cells (43).

#### Recovery or Chronic Myopathic Phase

Complete elimination of pathogens from the inflamed myocardium usually restores cardiac function. In genetically susceptible individuals, however, the breakdown of heart-specific tolerance and expansion of CD4+ effector T cells lead to chronic inflammation (8). Chronic myopathic phase is characterized by massive infiltration of CD4+ T cells, macrophages and antibodies (4, 8, 47), and the heart undergoes cardiac remodeling resulting in the progression into DCM and end-stage heart failure (48, 49).

As they are an integral part in subacute and chronic phases, a better understanding of dysregulated CD4+ T cells in the pathogenesis of myocarditis is called for. Moreover, modulating CD4+ T cell-related autoimmunity is the basis for a promising therapeutic strategy. Recent studies have identified that miRNAs regulate the pathogenesis of autoimmune diseases through the modulation of CD4+ T cell differentiation (50). In addition, miRNAs can epigenetically regulate cardiac function (22, 51), and can be manipulated by agonists or antagonists. Therefore, analyzing dysregulated miRNAs together with their mRNA and protein targets in heart biopsies and body fluids offer an opportunity to find potential therapeutic targets, though the crosstalk between miRNAs and CD4+ T cells in myocarditis is elusive.

## CD4+ T Cells in the Pathogenesis of Myocarditis

CD4+ T cells are key modulators in myocarditis, and the effector and regulatory subsets, respectively, promote and inhibit autoimmune responses. In addition, reciprocal regulation of CD4+ T cell subsets is also important in the pathogenesis of myocarditis.

### CD4+ T Cell Subsets and Related Cytokines in Myocarditis

Dysregulated CD4+ T cells and their related cytokines are critical for the development and progression of myocarditis (Table 1). In fact, all CD4+ T cell subsets are potentially cardiotoxic depending on their polarization (72), a process that depends on the integrated signals from TCRs, co-stimulatory receptors and cytokines. TCRs and co-stimulatory molecules determine the antigenic specificity of T cells, whereas the cytokines direct their lineage-specific differentiation (17, 46, 73–77).

**Table 1:** Dysregulated CD4+ T cells in the pathogenesis of myocarditis.

**CD4+T cells**	**Cytokines**	**Roles in the pathogenesis of myocarditis and relevant researches**	**References**
Th1 cells		Initiate tissue damage and protect the myocardium from excessive inflammation	(8, 10, 11)
	IFN-γ	Promote the early responses	
		In the sera of patients with acute myocarditis: high levels of IFN-γ were detected	(52)
		In the endomyocardial biopsies of patients with DCM: genes of IFN-γ were overexpressed	(53)
		In a TCR transgenic mouse model of EAM: knocking out IFN-γ receptor or inhibiting its downstream signaling pathway attenuated myocarditis	(54)
		In mice with VMC: depletion of IFN-γ during acute infection reduced myocarditis without affecting viral replication	(55, 56)
		In mice infected with *T. cruzi*: lowering IFN-γ production reduced myocarditis	(57)
		Protect from excessive inflammation	
		In mice with VMC: IFN-γ deficiency led to increased cardiac inflammation	(10, 11)
	TNF-α, IL-1β	In mice with VMC: increased production of IL-1β and TNF-α induced myocarditis	(58)
	IL-12	IL-12 receptor β1-KO mice were resistant to myocarditis induction, which was exacerbated in the wild-type EAM mice treated with exogenous IL-12	(36)
Th2 cells		Pathogenic in severe myocarditis with eosinophil expansion	(13)
		In the heart samples of patients with severe myocarditis: increased levels of Th2 cells and related cytokines were detected	(12)
		In a patient with acute eosinophilic myocarditis: anti-allergic Th2 cytokine inhibitor ameliorated cardiac inflammation	(59)
		In vitamin D receptor-KO mouse model: spontaneous Th2-biased inflammation was observed	(60)
		In mice with EAM: a Th2-biased phenotype was detected	(61)
	IL-4	In mice with EAM: administration of anti-IL-4 mAb significantly reduced disease severity and Th2 response	(61)
		In mice infected with *B. spirochetes* or *T. cruzi*: IL-4 deficiency exacerbated cardiac inflammation	(62, 63)
Th17 cells		Major regulators in late or chronic phase of myocarditis	(8, 16)
	IL-17	Play a critical role during cardiac remodeling, and is essential for the progression from myocarditis to DCM	
		In a clinical trial on 41 patients with acute myocarditis/DCM and 32 healthy volunteers: the proportion of circulating Th17 cells was significantly elevated in the patient group. In addition, increased Th17 cells were correlated with heart failure, and biopsies with detectable IL-17A+ cells showed greater fibrosis	(21)
		In the IL-12p35 and IL-12p40 knockout mouse models: the neutralization of IL-17 decreased the severity of myocarditis and cardiac autoantibody responses	(64)
		In IFN-γ-deficient mice: knocking out IL-17A did not ameliorate the severity of myocarditis, and the IL-17-deficient mice developed almost the same degree of myocarditis as the wild-type controls	(14)
		In IL-17A–deficient mice: myocardial fibrosis is reduced, and administering anti-IL-17A mAb to mice with established myocarditis reduced cardiac fibrosis and preserved ventricular function	(14)
Treg cells		Induce and maintain peripheral tolerance, and prevent excessive immune responses and autoimmunity	
		In acute myocarditis/DCM patients: circulating Treg cells were significantly decreased	(21)
		In nude mice: depletion of Treg cells led to the spontaneous development of fatal autoimmune myocarditis	(19)
		In mice with VMC: depletion of Treg cells aggravated cardiac fibrosis	(20)
		In mice infected with *T. cruzi*: mortality rates increased significantly after administering anti-CD25 or anti-GITR antibodies	(65)
		In mice with VMC: adoptive transfer of Treg cells prior to CVB3 infection attenuated excessive inflammatory response to the virus and facilitated viral clearing	(41)
		In mice with VMC: adoptive transfer of Treg cells after CVB3 infection significantly reduced cardiac fibrosis	(20)
		In a mouse model of chronic Chagas cardiomyopathy: recruitment of Treg cells decreased parasitic load and alleviated myocarditis	(66)
		In rat with EAM: *in vivo* Treg cell expansion decreased the severity of myocarditis	(67)
		Pathogenic in myocarditis.	
		In mice with VMC: PM2.5 exposure prior to CVB3 infection increased the proportion of Treg cells, and increased the severity of myocarditis	(68)
		Ambiguous role in fibrosis	
		In mice with VMC: adoptive transfer of Treg cells into mice after CVB3 infection significantly reduced cardiac fibrosis via IL-10 secretion	(20)
		In a mouse model of chronic heart failure: Treg cells secreted high levels of TGF-β and only miniscule amounts of IL-10, which stimulated cardiac fibrosis	(69)
		Strain- and gender-specific variations in myocarditis susceptibility	(70, 71)

#### Th1 Cells and Related Cytokines in Myocarditis

Th1 cells act as a two-edged sword in myocarditis, and not only initiate tissue damage but also protect the myocardium from excessive inflammation (8, 10, 11). They promote the early responses in myocarditis via pro-inflammatory cytokines such as interferon (IFN)-γ, tumor necrosis factor (TNF)-α, interleukin (IL)-1β and IL-12 (8), and IFN-γ is the signature cytokine of this subset (16).

High levels of IFN-γ were detected in the sera of patients with acute myocarditis (52), and genes of IFN-γ were overexpressed in the endomyocardial biopsies of patients with DCM (53). Consistent with this, in a TCR transgenic mouse model of experimental autoimmune myocarditis (EAM), knocking out IFN-γ receptor and/or inhibiting its downstream signaling pathway significantly attenuated cardiac inflammation (54). Furthermore, IFN-γ was detected in the hearts of mice with viral myocarditis (VMC), and its depletion during acute infection reduced cardiac inflammation without affecting viral replication (55, 56). Likewise, in mice infected with *T. cruzi* lowering IFN-γ production reduced symptoms of myocarditis (57). These findings indicate that IFN-γ acts as a promoter in the initiation of myocarditis and Th1 cells play a crucial role in myocarditis. However, some studies on murine myocarditis models showed that IFN-γ deficiency leads to increased cardiac inflammation (10, 11). This paradox may be explained by the findings in mice with EAM that IFN-γ signaling promoted monocyte differentiation into the regulatory nitric oxide (NO)–producing DCs, and NO in turn could limit expansion of the antigen-specific T-cells (11).

Other Th1-related pro-inflammatory cytokines like IL-1β, TNF-α, and IL-12 play pathogenic roles in myocarditis as well. Increased production of IL-1β and TNF-α in response to CVB3 virus infection induced myocarditis in a mouse model (58). Similarly, the IL-12 receptor β1-KO mice were resistant to myocarditis induction, which was exacerbated in the wild-type EAM mice treated with exogenous IL-12 (36).

#### Th2 Cells and Related Cytokines in Myocarditis

Th2 cells and related cytokines like IL-4 and IL-13 are critical in severe myocarditis with eosinophil expansion (12, 13), and IL-4 is the signature cytokine (78).

Increased levels of Th2 cells and related cytokines were detected in the hearts of patients with myocarditis and advanced heart failure, compared to patients with DCM (12). Furthermore, an anti-allergic Th2 cytokine inhibitor ameliorated cardiac inflammation in a patient with acute eosinophilic myocarditis (59), underscoring the critical role of Th2 cells in myocarditis. Spontaneous Th2-biased inflammation was observed in the myocardium of the vitamin D receptor-KO mouse model (60). Similarly, a Th2-biased phenotype was also detected in A/J mice with EAM, with infiltration of eosinophils and giant cells in the heart and increased levels of total IgE antibodies (61). Administration of anti-IL-4 monoclonal antibody (mAb) significantly reduced disease severity as well as the Th2 response, suggesting that IL-4 is a key pathogenic factor (61). However, IL-4 deficiency exacerbated cardiac inflammation in infectious models of myocarditis induced by *Borrelia spirochetes* or *T. cruzi*, which could be due to a compensatory increase in the Th 1 response in these mice (62, 63).

#### Th17 Cells and Related Cytokines in Myocarditis

Th17 cells and related cytokines like IL-17A, IL-17F, IL-22, TNF-α are the major regulators of the late or chronic phase of myocarditis (8, 14–16), and IL-17A and IL-17F are signature cytokines.

The neutralization of IL-17 in the IL-12p35 and IL-12p40 knockout mouse models decreased the severity of myocarditis and cardiac autoantibody responses, suggesting that IL-17 is critical for EAM (64). However, knocking out IL-17A did not ameliorate the severity of myocarditis in IFN-γ-deficient mice, and the IL-17-deficient mice developed almost the same degree of myocarditis as the wild-type controls, suggesting that Th17 is dispensable in acute myocarditis (14). Furthermore, myocardial fibrosis was reduced in IL-17A–deficient mice, and administering anti-IL-17A mAb to mice with established myocarditis reduced cardiac fibrosis and preserved ventricular function (14). Subsequent studies showed that IL-17A-driven inflammatory DCM is mediated through cardiac fibroblasts (15). Thus, IL-17A plays a critical role during cardiac remodeling, and is essential for the progression from myocarditis to DCM. In a clinical trial on 41 patients with acute myocarditis/DCM and 32 healthy volunteers, the proportion of circulating Th17 cells was significantly elevated in the patient group (21). Furthermore, increased Th17 cells are correlated with heart failure, and biopsies with detectable IL-17A+ cells show greater fibrosis (21). These findings confirmed that Th17 cells play critical roles in myocarditis and its progression into DCM, and cardiac myosin-Th17 responses increase the risk of heart failure in patients with myocarditis.

#### Treg Cells and Related Cytokines in Myocarditis

Treg cells are necessary for the induction and maintenance of peripheral tolerance, as well as prevention of excessive immune responses and autoimmunity. In myocarditis, Treg cells relieve acute cardiac inflammation and prevent progression from myocarditis to DCM (16–21). This subset expresses Fork head box protein 3 (FOXP3) and CD25, and suppresses effector T cells either via anti-inflammatory cytokines like IL-10, IL-35, and TGF-β, or immunosuppressive receptors like CTLA-4 and glucocorticoid induced tumor necrosis factor receptor (GITR) (16, 17).

Mutations in the FOXP3 gene elicit severe autoimmune, inflammatory, and allergic responses in multiple organs in both humans and rodents (18). Depletion of Treg cells in nude mice by adoptive transfer of GITR-deficient CD4+ T cells led to the spontaneous development of fatal autoimmune myocarditis (19). Consistent with this, depletion of Treg cells in the CVB3-infected mice by injecting anti-CD25 mAb aggravated cardiac fibrosis (20). Likewise, the mortality rates of mice infected with *T. cruzi* increased significantly after administering anti-CD25 or anti-GITR antibodies (65). Circulating Treg cells were also significantly decreased in patients with acute myocarditis/DCM compared to healthy volunteers (21). However, myocarditis was not observed in a mouse model of IPEX (immune dysregulation, polyendocrinopathy, enteropathy, X-linked syndrome), a rare autoimmune disease characterized by congenitally dysfunctional Treg cells due to FOXP3 mutations (18). This could be due to the inter-species differences or multiple mechanisms regulating Treg cell functions, and needs further investigation. Nevertheless, adoptive transfer of Treg cells prior to CVB3 infection not only attenuated the excessive inflammatory response to the virus but also facilitated viral clearing from the murine myocardium (41). Furthermore, adoptive transfer of isolated Treg cells into mice after infection with CVB3 significantly reduced cardiac fibrosis (20). Similarly, recruitment of Treg cells decreased parasitic load and alleviated myocarditis in a mouse model of chronic Chagas cardiomyopathy (66), and *in vivo* Treg cell expansion via adenovirus-mediated CTLA4Ig gene therapy decreased the severity of myocarditis in a rat model of EAM (67).

However, some studies have demonstrated a causative role of Treg cells in myocarditis. For example, particulate matter (PM) 2.5 exposure prior to CVB3 infection increased the proportion of Treg cells in mice, and increased the severity of myocarditis (68). It is possible that PM2.5-induced Treg cell expansion suppressed anti-viral immune responses, which aggravated virus-mediated cardiac injury. Furthermore, long-term activation of Treg cells could lead to the loss of CD4+ T cell immune function and promote myocarditis.

In addition, the role of Treg cells in fibrosis is also ambiguous. Adoptive transfer of isolated Treg cells into mice after CVB3 infection significantly reduced cardiac fibrosis via IL-10 secretion (20). However, Treg cells secrete the pro-fibrotic TGF-β, and suppression of TGF-β can alleviate cardiac fibrosis. In a mouse model of chronic heart failure, Treg cells secreted high levels of TGF-β, and only miniscule amounts of IL-10, which stimulated cardiac fibrosis (69). These conflicting results could be due to differences in the number of circulating Treg cells, which might be responsible for the strain- and gender-specific variations in myocarditis susceptibility (70, 71).

#### Novel CD4+ T Cell Subsets

Recently discovered CD4+ T cell subsets include Th9, Th22, and T follicular helper (Tfh) cells, etc. They show distinct effector fates that are independent of programs of subsets mentioned above (79). The potential roles of these novel subsets in cardiac inflammation and autoimmunity remain to be elucidated.

### Interactions of CD4+ T Cell Subsets in Myocarditis

The roles of CD4+ T cell subsets in myocarditis greatly depend on the interactions between the resident and infiltrating inflammatory cell types. The reciprocal interplay of distinct CD4+ T cell subsets has only been partly deciphered (17).

#### Th1/Th2 Balance

Researches have shown that a systemic Th1/Th2 imbalance exist during the clinical phase of myocarditis. Analysis of peripheral CD4+ T cells in a patient with acute myocarditis demonstrated that Th1 was dominant during the acute phase and coincided with increased serum levels of creatine kinase (CK), while Th2 predominated during the recovery phase and was accompanied by decreased CK levels (80). Consistent with this, restoring the Th1/Th2 inflammatory cytokine balance hindered the progression of autoimmune myocarditis (81). Interestingly, Huber et al. found that the immune response to CVB3 in male and female mice is mediated by the Th1 and Th2 cells, respectively (82). A prerequisite to the Th1/Th2 model is mutual antagonism of the two phenotypes at the clonal level. However, the discovery of novel CD4+ T cell subsets and the heterogeneity and plasticity among CD4+ T cell subsets contradict the binary nature of Th1/Th2 model and portend more complex interactions (83).

#### Th1/Th17 Balance

The balance between Th1 and Th17 cells determines the transition from myocarditis to DCM. In a TCR transgenic mouse model with spontaneous autoimmune myocarditis progressing into DCM, blocking IFN-γ signaling significantly attenuated myocarditis without affecting the development of DCM, while lack of IL-17A inhibited DCM but had a mild effect on myocarditis (54). In addition, cardiac IFN-γ production was significantly lower in the IL17a-KO mice compared to the wild-type mice with EAM (14). In contrast, activation of naive CD4+ T cells in the presence of exogenous IL-17 or IL-17F did not lead to Th17 polarization regardless of IFN-γ (84), suggesting that Th17 differentiation likely affects Th1 cell functions.

#### Th17/Treg Balance

Most studies implicate Th17 and Treg cells with, respectively, detrimental and beneficial effects in myocarditis, and a lower proportion of Treg cells has been associated with a stronger Th17 response and more severe autoimmune myocarditis (70). A recent study showed that ectopic expression of TAZ, a critical co-activator of the Th17-defining transcription factor RORγt, or depletion of TAZ-inhibitor increased the proportion of Th17 cells and decreased that of Treg cells. In contrast, mice with TAZ-deficient T cells had more Treg cells and fewer Th17 cells (85). However, the number of splenic Th17 and Treg cells, as well as the levels of related cytokines increased significantly in the mice infected with CVB3, and neutralization of IL-17 decreased the proportion of Treg cells (86). The exact function of both subsets likely depends on whether the cardiac injury is a direct consequence of viral infection or an indirect immunopathological effect, since inhibition of Treg-induced anti-viral immunity can lead to a persistent viral infection, while Th17 cells may protect against viral infections (87).

Since CD4+ T cells paly central roles in myocarditis and orchestrate the disease, they are a viable therapeutic target. Recent studies have identified that miRNAs can modulate CD4+ T cell activation and differentiation (88–92), and can regulate the pathogenesis of autoimmune diseases through the modulation of CD4+ T cell differentiation (50). The modulation of these miRNAs offers great opportunities for developing novel strategies to treat patients with myocarditis. However, the crosstalk between miRNAs and CD4+ T cells in myocarditis is elusive. Thus, dysregulated miRNAs in myocarditis need to be further investigated.

## Dysregulated miRNAs in Myocarditis

**Figure 2:**
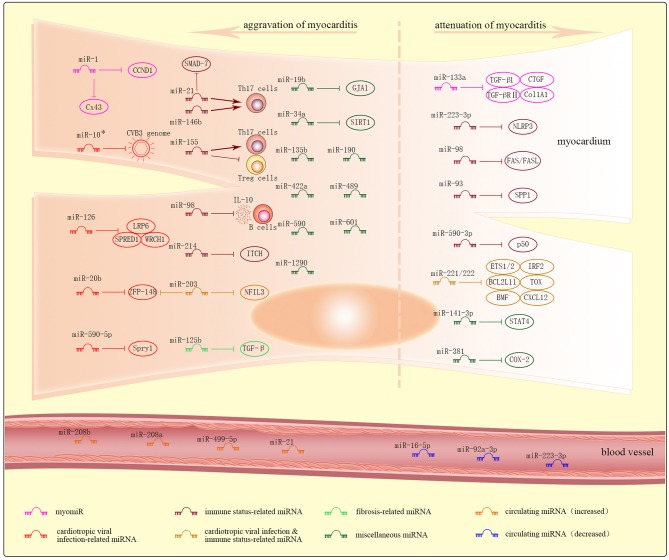
Dysregulated miRNAs and their targets in myocarditis. Dysregulated intracellular miRNAs in myocarditis are further and roughly classified into myomiRs, cardiotropic viral infection-related miRNAs, immune status-related miRNAs, cardiotropic viral infection and immune status-related miRNAs, fibrosis-related miRNAs, and miscellaneous miRNAs. Most of these miRNAs result in the aggravation of myocarditis, while others result in the attenuation of myocarditis. Increased or decreased levels of circulating miRNAs can be detected in plasma of patients with myocarditis.

miRNAs are a highly conserved class of endogenous single-stranded non-coding RNA molecules, ~19–25 nucleotides in length. Their regulatory function was first described in *Caenorhabditis elegans* in 1993 (93). Since then, numerous miRNAs have been identified with specific target genes and biological functions. miRNA profiles of myocarditis have been analyzed (23), and dysregulated miRNAs in myocarditis along with their mRNA and protein targets have been identified in heart biopsies (intracellular miRNAs) and body fluids (circulating miRNAs) (Figure 2). Several dysregulated miRNAs in myocarditis are involved in the etiology and pathogenesis of myocarditis, show phase-dependent changes, and correlate with viral infection, immune status, fibrosis, destruction of cardiomyocytes, arrhythmias, cardiac functions, and outcomes. Thus, miRNAs are promising diagnostic markers and therapeutic targets in myocarditis.

### Biogenesis and Functions of miRNAs

miRNAs are commonly located in intergenic regions, introns and polycistronic sites, and are transcribed either in clusters or individually (94). Biogenesis and maturation of miRNAs have been extensively reviewed (95–98). The primary miRNAs (pri-miRNAs) transcribed by polymerase II have a stem-loop structure (5′ cap and a 3′ poly A tail) similar to that in mRNAs. They are processed by RNase III Drosha into smaller 60–70 bp hairpin precursor miRNAs (pre-miRNAs), which are then transported from the nucleus to the cytoplasm through Exportin 5 and cleaved by the RNase III Dicer into short mature miRNA duplexes consisting of a guide strand and passenger strand. These duplexes bind to the Argonaute (Ago) protein to form a mature RNA-induced silencing complex known as RISC. During RISC assembly, the two strands of the miRNA duplex are separated, and the passenger strand is discharged from Ago and degraded, leaving the guide strand to bind to the target mRNA. However, recent studies show that pri-miRNAs can also be transcribed by RNA polymerase III (99), and certain debranched introns that structurally mimic pre-miRNAs can also enter the miRNA-processing pathway (100). In addition, there is evidence that passenger strands can also be incorporated into RISC and trigger gene silencing (101, 102).

Following their biogenesis, the mature miRNA is capable of post-transcriptionally inducing gene silence through its seed region, which is located at nucleotides 2–7 at the 5′ end and acts as the main parameter for binding to the target mRNAs (103). In most cases, the seed region binds to the 3′ untranslated region (UTR), but in some cases it can also bind to the 5′ UTR or even the coding region of the targets (103–105). Depending on the degree of complementarity, and the length and accessibility of the binding sites, miRNAs can either inhibit translational expression or promote the degradation process (106, 107). However, recent studies have shown that some miRNAs may unconventionally enhance rather than suppress gene transcription (108). A single miRNA affects multiple mRNAs, and a single mRNA can also be regulated by different miRNAs (104). miRNAs regulate more than 60% of human protein-coding genes (97).

Proper functioning of the heart requires a delicate balance between chemical and electrical stimuli that control cardiac muscle contraction (109), and miRNAs are ideal regulators to exert the control over these synchronous processes (22). In fact, miRNAs are critical epigenetic regulators of cardiac function and modulate almost all aspects of cardiac physiology and pathology (22). Ablation of essential components of miRNA biogenesis results in severe developmental defects in the cardiovascular system (30, 31). The function of miRNAs in myocarditis depends on the temporal and spatial expression of their targets. For example, miR-155 increases the production of inflammatory cytokines and activates immune effector cells in the heart during infection, but switches to an anti-inflammatory and cardioprotective role during sepsis (110). Similarly, miR-21 levels correlate positively with the severity of myocarditis (111, 112), although one study showed opposing result that injecting miR-21 to mice with VMC relieves disease severity (113).

### miRNAs and CD4+ T Cells

miRNAs are important players in T cell differentiation and function. Absence of miRNAs during the early developmental stage of T cells adversely affects thymocyte survival (92, 114), while that at late developmental stages decreases the number of peripheral CD4+ T cells and blocks CD8+ T cell development (92, 115).

Inhibition of all miRNAs in CD4+ T cells skews them toward Th1 as opposed to Th2 differentiation, and reduces the number of Treg cells (115–117). Several specific miRNAs have also been identified that control the differentiation and function of distinct CD4+ T cell subsets. For example, miR-21 regulates Th1 cell differentiation by modulating IL-12 production, and maintains the Th1/Th2 balance (118). Deficiency of miR-21 enhances Th1 cell development *in vivo* (118), and its overexpression in T cells induces Th2 cell differentiation *in vitro* (119). In addition, miR-21 promotes Th17 differentiation by targeting SMAD-7, a negative regulator of TGF-β signaling (89). Highly expressed in Treg cells, miR-155 promotes the maintenance of Treg cells, and enhances both Th1 and Th17 cell-dependent tissue inflammation (120). miR-155 deficiency decreases T cell-dependent humoral and cellular immune responses (120, 121), and induces Th2 polarization *in vivo* and *in vitro* (122, 123).

### Intracellular miRNAs in Myocarditis

In this review, dysregulated intracellular miRNAs identified in heart biopsies in myocarditis are further and roughly classified into myomiRs, cardiotropic viral infection-related miRNAs, immune status-related miRNAs, cardiotropic viral infection and immune status -related miRNAs, fibrosis-related miRNAs, and miscellaneous miRNAs.

#### MyomiRs

MyomiRs, or miRNAs expressed predominantly in cardiac tissues, correlate with endomyocardial inflammation, cardiac fibrosis, destruction of cardiomyocytes, arrhythmias, cardiac functions, and outcomes in myocarditis. These miRNAs mainly include miR-1, miR-133a, miR-208a, miR-208b, miR-499, etc.

miR-1 is the most abundant myomiRs, accounting for ~40% of all sequenced cardiac miRNAs (124), and miR-133 is transcribed along with miR-1 from the same bicistronic transcript (125). The both miRNAs regulate cardiomyocyte differentiation and proliferation during development by synergistically targeting numerous genes. During cardiomyocyte maturation, however, miR-1 and miR-133 work antagonistically and, respectively, promote and inhibit the process (126). They are also involved in cardiomyopathies like myocarditis and congenital heart diseases (32, 127–129). In a mouse model of VMC, miR-1 promoted the destruction of cardiomyocytes and triggered arrhythmia by repressing Connexin 43 (Cx43), the major gap-junction protein expressed in the heart modulating proper function of the cardiac conduction system (127). In addition, miR-1 is also a key element in chronic Chagas disease cardiomyopathy by controlling cyclin D1 (CCND1), a positive cell cycle regulator in the proliferation of cardiomyocytes during normal heart development (129). In a study of patients with inflammatory cardiomyopathy, miR-133a attenuated both endomyocardial inflammation and fibrosis possibly by repressing transforming growth factor-β1 (TGF-β1), TGF-β receptor type II (TGF-βRII) (130), connective tissue growth factor (CTGF) (131), or collagen 1a1 (Col1A1) (132), and correlated with improved left ventricular ejection function and better prognosis (128). The miR-208 family consisting of miR-208a, miR-208b, and miR-499 direct cardiac myosin gene expression since they are transcribed from the introns of genes encoding the main components of muscle fibers, such as Myh6, Myh7, and Myh7b (133). So far few researches have shown the roles of the miR-208 family in myocarditis other than in other cardiac pathologic conditions, though they have been detected in the plasma of patients with acute myocarditis (134, 135).

#### Cardiotropic Viral Infection-Related miRNAs

Cardiotropic viral infection-related miRNAs regulate viral replication or virulence either by directly targeting the virus genome, or indirectly by targeting the host genes that can regulate responses to viral infection. These miRNAs mainly include miR-10^*^, miR-20b, miR-126, miR-590-5p, etc.

miR-10^*^ targeted the CVB3 genome to enhance viral biosynthesis during myocarditis, and was detectable in the cardiac tissues of suckling BALB/c mice (136). In mice with VMC, miR-20b enhanced viral replication by targeting zinc finger protein-148 (ZFP-148), a inhibitory factor in the regulation of virus replication (137). In cardiomyocyte HL-1 cells infected with CVB3, miR-126 facilitated viral replication by suppressing sprouty-related, EVH1 domain containing 1 (SPRED1), low-density lipoprotein receptor-related protein 6 (LRP6), and Wnt-responsive Cdc42 homolog 1 (WRCH1), inhibitory factors to the viral cytopathogenicity (138), and miR-590-5p facilitated prolonged viral replication by suppressing antiviral sprouty-1 (Spry1), moreover, cells over-expressing miR-590-5p were more susceptible to infection (139).

#### Immune Status-Related miRNAs

Immune status-related miRNAs regulate multiple aspects of the inflammatory response by binding to immune-based targets. These miRNAs mainly include miR-223-3p, miR-21, miR-146b, miR-155, miR-98, miR-93, miR-590-3p, miR-214, etc.

In mice with EAM, miR-223-3p ameliorated inflammatory response by targeting pyrin domain-containing-3 (NLRP3) inflammasome which is a multiprotein complex and sensor in innate immune cells (140).

miR-21, miR-146b, and miR-155 are consistently up-regulated in patients with acute VMC (141), and mice with myocarditis induced by CVB3 or *T. cruzi* (112). Consistent with this, intravenous injection of miR-21 and -146b antagonists decreased the expression levels of Th17 and RORγt, and attenuated cardiac inflammation and myocardial damage in a murine model of VMC (111). Likewise, miR-155 antagonists led to Th17/Treg imbalance by reducing Th17 response and the secretion of Th17-polarizing cytokines in a mouse model of EAM, and attenuated disease severity and cardiac injury (142).

miR-98 exacerbated myocarditis by suppressing IL-10 expression in B cells in mice with EAM (143). In contrast, miR-98 could attenuate myocardial cell apoptosis by targeting FAS/FASL (144).

Suppressing secreted phosphoprotein 1 (SPP1) and p50 can inhibit NF-κB pathways, while suppressing ITCH, an NF-κB signaling suppressor, can enhance NF-κB pathways. Consist with this, overexpression of miR-93 in mice with VMC or miR-590-3p in rats with EAM reduced cardiac inflammation by targeting SPP1 or p50, respectively (145, 146), while overexpression of miR-214 in neonatal murine cardiac myocytes infected with VMC enhanced myocardial inflammation by targeting ITCH (147).

#### Cardiotropic Viral Infection and Immune Status-Related miRNAs

Cardiotropic virulence & inflammation-related miRNAs orchestrates the antiviral and inflammatory immune response. These miRNAs mainly include miR-221/-222 family, miR-203, etc.

In mice with VMC, miR-221/-222 targeted ETS1/2, IRF2, BCL2L11, TOX, BMF, and CXCL12 to coordinate the increased viral replication and inflammation (148). Systemic inhibition of miR-221/-222 family *in vivo* increased cardiac viral load, prolonged the viremic state, and aggravated cardiac inflammation and injury; while its upregulation decreased viral replication (148) and protected H9c2 cells against LPS-induced injury (149). In mice with VMC, miR-203 enhanced viral replication by suppressing zinc finger protein-148 (ZFP-148), an inhibitory factor in the regulation of virus replication (150). Furthermore, in LPS-treated cardiomyocyte H9c2, miR-203 accelerated apoptosis and inflammation by suppressing nuclear factor interleukin-3 (NFIL3) (151).

#### Fibrosis-Related miRNAs

Fibrosis-related miRNAs modulate myocardial fibrosis, and mainly include miR-133a (discussed in myomiRs), miR-21, miR-125b, etc.

In a mouse model of chronic VMC, miR-21 promoted the myocardial fibrosis in chronic VMC possibly by suppressing SMAD-7, a negative regulator of TGF-β signaling (152). In mice with EAM, inhibition of miR-125b targeting TGF-β attenuated cardiac fibrosis (153).

#### Miscellaneous miRNAs

Miscellaneous miRNAs identified in myocarditis show distinct functions in myocarditis and cannot be classified into any of the above types. These miRNAs mainly include miR-19b, miR-34a, miR-141-3p, miR-381, etc.

miR-19b contributed to cardiac arrhythmia through repression of gap junction protein α1 (GJA1) in a mouse model of VMC (154). miR-34a promoted cardiomyocyte apoptosis by downregulating sirtuin 1 (SIRT1), an inhibitory factor to p53-dependent apoptosis (155). miR-141-3p alleviated the inflammatory response by inhibiting signal transducer and activator of transcription 4 (STAT4) in mice with EAM (156). miR-381 alleviated myocardial cell injury by inhibiting inflammatory factor cyclooxygenase-2 (COX-2) in mice with VMC (157). In addition, miR-155, miR-135b, miR-190, miR-422a, miR-489, miR-590, miR-601, and miR-1290 were strongly induced in the hearts of patients with late viral persistence and progressive cardiac dysfunction (158).

Since the agonist/antagonist of dysregulated miRNAs can potentially control the severity of disease, miRNAs are potential therapeutic targets in myocarditis. However, most *in vivo* miRNA studies conducted so far have focused on site-specific phenotypic rather than systemic effects, and might ignore the off-target effects (159). Other challenges at present are to improve miRNA delivery and selective targeting of cardiac mRNAs, maximize efficiency with lowest doses, minimize adverse effects, and achieve competent pharmacokinetics and pharmacodynamics. Furthermore, large-scale clinical application of miRNA-based therapy is currently impeded by the costs of production, storage, and lack of oral delivery methods.

### Circulating miRNAs in Myocarditis

miRNAs can not only modulate target mRNAs intracellularly, but also participate in intercellular communication via extracellular vesicles (EVs) like exosomes, microvesicles, and apoptotic bodies (156), or as part of protein/lipoprotein complexes (160). Following a stressful stimulus, miRNAs can be released in an active (secretion) or passive (membrane leaking) manner from various tissues/organs into the body fluids, thereby constituting the circulating miRNA fraction (161, 162). These miRNAs are remarkably stable, resistant to endogenous RNase degradation, and directly affect diverse physiological and pathological processes (161, 163). Dysregulated circulating miRNAs in myocarditis may correlate with disease severity, show phase-dependent changes and even predict prognosis. So far, no single miRNA has been identified as a specific marker for chronic myocarditis.

Corsten et al. detected increased levels of miR-208b and miR-499-5p in the plasma of 14 patients with acute VMC, which correlated positively with disease severity based on troponin T levels and ejection fraction (134). Furthermore, miR-21 and miR-208a were significantly increased during the acute phase (at admission) in 8 pediatric patients with VMC, and the levels of miR-208a during the subacute phase (9.3 ± 3.4 days from admission) correlated with the degree of recovery of the left ventricular ejection function during the resolution/chronic phase (100.8 ± 35.4 days from admission) (135). However, neither study directly compared miRNA levels with troponin values to identify the superior prognostic marker, and these miRNAs are also elevated in other cardiomyopathies (134). In addition, pediatric patients with rheumatic carditis showed markedly decreased levels of miR-16-5p, miR-92a-3p, and miR-223-3p in plasma (164).

Since the levels of circulating miRNAs correlate with disease severity, can predict the clinical outcome, and almost accord with the criteria for the candidate markers which include high specificity and sensitivity, ease of detection, low costs, and biological relevance (165, 166), they are potential diagnostic or prognostic markers. However, the results concluded by different observers have been conflicting, and the related studies have rather low reproducibility. These discrepancies are due to variations in sample size, technical aspects (such as sample collection, sample preparation, RNA isolation, endogenous miRNA normalization panels, and miRNA quantification methods), and lack of standard cut-off values (22, 167).

## Conclusions and Perspectives

Myocarditis is a polymorphic disease complicated with indeterminate etiology and pathogenesis. Both dysregulated CD4+ T cells and miRNAs play key roles in the pathogenesis of myocarditis. CD4+ T cells are key modulators, and the effector and regulatory subsets, respectively, promote and inhibit autoimmune responses. In addition, the reciprocal interplay of CD4+ T cell subsets influence pathogenesis as well. Causal links between dysregulated miRNAs and myocarditis have been supported by recent epigenetic researches, and dysregulated miRNAs along with their mRNA and protein targets have been identified in heart biopsies and body fluids during myocarditis. These miRNAs show phase-dependent changes, and correlate with viral infection, immune status, fibrosis, destruction of cardiomyocytes, arrhythmias, cardiac functions, and outcomes. Recent developments in the field of CD4+ T cells and miRNAs in myocarditis have advanced our understandings, which can be translated to reliable diagnostic markers and effective miRNA-based therapy.

However, the precise and accurate roles of dysregulated CD4+ T cells and miRNAs in myocarditis as well as the crosstalk between them remain far from being fully understood. In addition, novel CD4+ T cell subsets and miRNAs involved in myocarditis remain to be identified. Guidelines on the most appropriate design and analysis methods to increase replicability of studies on circulating miRNAs are scarce. Likewise, more multicenter trials in large cohorts are required to confirm the reliability of miRNAs as diagnostic markers and therapeutic targets. As for therapeutic applications, the systemic effects of the candidate miRNAs have to be investigated further in order to avoid off-target effects. In addition, novel delivery strategies, reduced cost of production, and storage remain areas of concern.

## Author Contributions

JW reviewed the literature and was responsible for writing the manuscript. BH revised the manuscript.

### Conflict of Interest

The authors declare that the research was conducted in the absence of any commercial or financial relationships that could be construed as a potential conflict of interest.
